# Standardising visual control devices for Tsetse: East and Central African Savannah species *Glossina swynnertoni*, *Glossina morsitans centralis* and *Glossina pallidipes*

**DOI:** 10.1371/journal.pntd.0006831

**Published:** 2018-09-25

**Authors:** Mechtilda Byamungu, Tusevo Zacarie, Alexis Makumyaviri M’Pondi, Philémon Mansinsa Diabakana, Andrew McMullin, Thomas Kröber, Steve Mihok, Patrick M. Guerin

**Affiliations:** 1 Tsetse and Trypanosomiasis Research Institute, Tanga, Tanzania; 2 Department of Pathology, Instituto de Investigação Veterinaria, Huambo, Angola; 3 School of Veterinary Medicine, The University of Lubumbashi, Lubumbashi, Democratic Republic of Congo; 4 Programme National de Lutte contre la Trypanosomiase Humaine Africaine (PNLTHA), Kinshasa/ Gombe, Democratic Republic of Congo; 5 Institute of Biology, Faculty of Science, University of Neuchâtel, Neuchâtel, Switzerland; 6 Independent scientist, Russell, Ontario, Canada; Makerere University, UGANDA

## Abstract

**Background:**

This study focused on the savannah tsetse species *Glossina swynnertoni* and *G*. *morsitans centralis*, both efficient vectors of human and animal trypanosomiasis in, respectively, East and Central Africa. The aim was to develop long-lasting, practical and cost-effective visually attractive devices that induce the strongest landing responses in these two species for use as insecticide-impregnated tools in population suppression.

**Methods and findings:**

Trials were conducted in different seasons and years in Tanzania *(G*. *swynnertoni)* and in Angola and the Democratic Republic of the Congo (DRC, *G*. *m*. *centralis*) to measure the performance of traps (pyramidal and epsilon) and targets of different sizes, shapes and colours, with and without chemical baits, at different population densities and under different environmental conditions. Adhesive film was used to catch flies landing on devices at the remote locations to compare tsetse-landing efficiencies. Landing rates by *G*. *m*. *centralis* in both Angola and the DRC were highest on blue-black 1 m^2^ oblong and 0.5 m^2^ square and oblong targets but were not significantly different from landings on the pyramidal trap. Landings by *G*. *swynnertoni* on 0.5 m^2^ blue-black oblong targets were likewise not significantly lower than on equivalent 1 m^2^ square targets. The length of target horizontal edge was closely correlated with landing rate. Blue-black 0.5 m^2^ targets performed better than equivalents in all-blue for both *G*. *swynnertoni* and *G*. *m*. *centralis*, although not consistently. Baiting with chemicals increased the proportion of *G*. *m*. *centralis* entering pyramidal traps.

**Conclusions:**

This study confirms earlier findings on *G*. *swynnertoni* that smaller visual targets, down to 0.5 m^2^, would be as efficient as using 1 m^2^ targets for population management of this species. This is also the case for *G*. *m*. *centralis*. An insecticide-impregnated pyramidal trap would also constitute an effective control device for *G*. *m*. *centralis*.

## Author summary

*Glossina swynnertoni* is restricted to open savannah in northwestern Tanzania and southwestern Kenya whereas *G*. *morsitans centralis* has a much wider distribution from western Tanzania/southern Uganda westwards through Zambia and southeast of the Democratic Republic of the Congo (DRC) to Angola. Both are savannah tsetse and are efficient vectors of human and animal trypanosomiasis. In comparison to other tsetse species, relatively little work has been done to test the efficacy of traps and targets for controlling *G*. *swynnertoni* and *G*. *m*. *centralis*. To determine the most visually-attractive and practical objects we conducted field tests with devices of various shapes, sizes and colours in Tanzania, DRC and Angola in different years, seasons, environmental conditions and at different population densities. The strongest landing responses were on 0.5 m^2^ horizontal rectangular targets with respect to ground that had both black and phthalogen blue elements with fly landing rates not significantly lower than on equivalent 1 m^2^ targets used till now for both species. The pyramidal trap proved efficient as a landing stimulus as targets of either size for *G*. *m*. *centralis*. Insecticide-impregnated blue-black 0.5 m^2^ cloth targets show promise as cost-effective devices for management of *G*. *swynnertoni* and *G*. *m*. *centralis* populations.

## Introduction

Diseases transmitted by tsetse flies, notably human African trypanosomiasis (HAT or sleeping sickness) and African animal trypanosomosis (AAT or Nagana), are caused by the transmission of trypanosomes, and are still a serious health and economic burden in sub-Saharan Africa [1,2]. After a resurgence in cases in the 1990s [3], increased treatment and vector control reduced the reported incidence of HAT from over 30,000 per year to below 3,000 per year in 2015 [1]. However, many more cases still go untreated, with an estimated 30,000 unreported cases in 2012 [3], and recalcitrant HAT foci remain across the continent [4]. Despite recent improvements, the economic and social cost of AAT continues to be a major burden in rural areas, where it is a significant cause of poverty and malnutrition [5]. This study focuses on *Glossina swynnertoni* Austen (Diptera, Glossinidae) and *G*. *morsitans centralis* Machado, two closely related savannah or *Morsitans* group tsetse [6]. Important information on *G*. *pallidipes* was also collected and is reported.

Both *G*. *swynnertoni* and *G*. *m*. *centralis* are efficient vectors of human and animal trypanosomiasis [7, 8] and HAT foci persist within the geographic ranges of these species [4]. Historically, in northern Tanzania, *G*. *swynnertoni* was found to have a higher trypanosome infection rate than *G*. *pallidipes* [7], but confirmation of infection with *T*. *brucei* required the use of special techniques [9]. Both species have since been the focus of several studies in the context of HAT cases in the Serengeti [10, 11, 12]. The trypanosome transmission capacity of *G*. *m*. *centralis* is equal to or greater than that of *G*. *pallidipes*, depending on the trypanosome species [8, 13].

**Fig 1:**
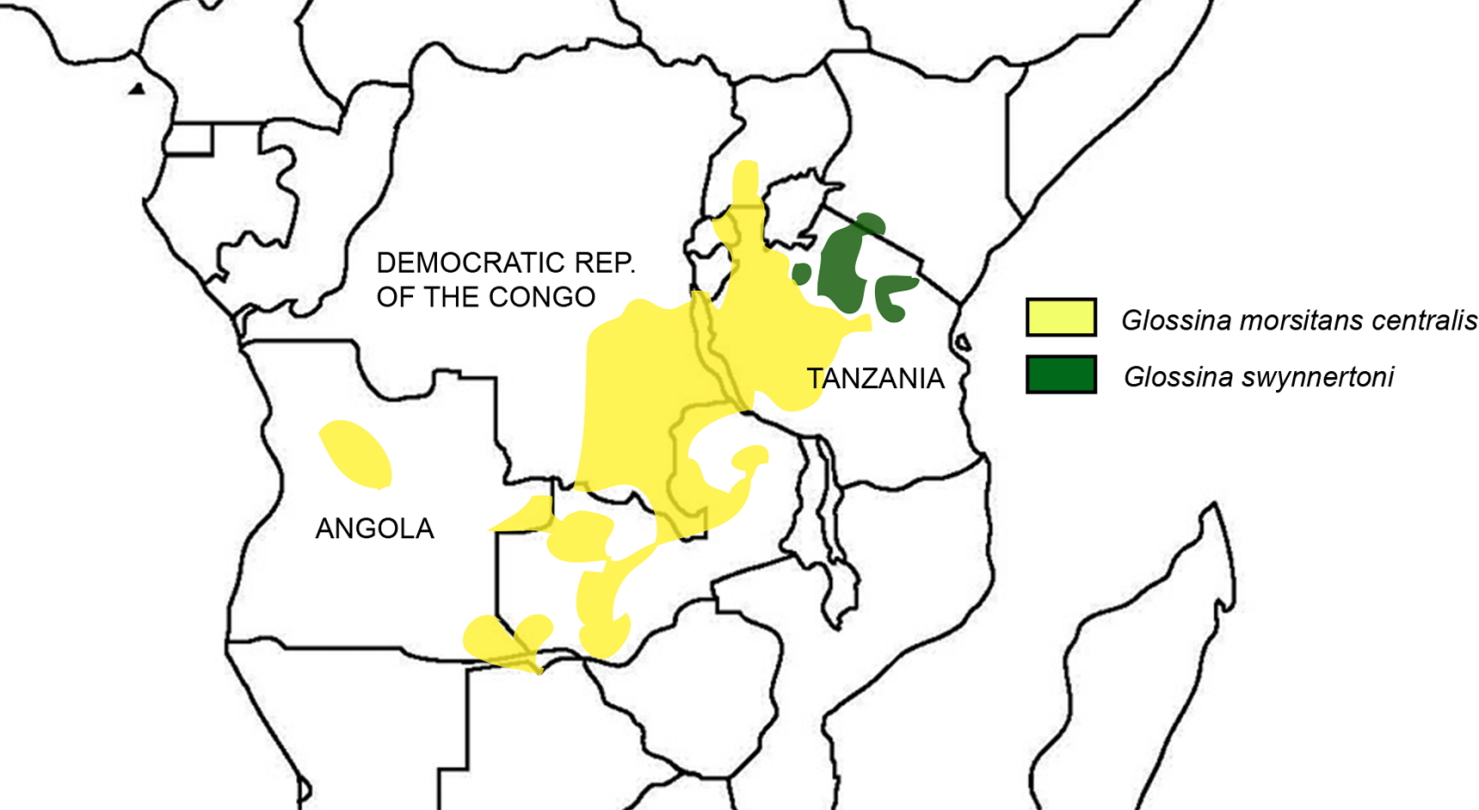
Outlines of the main distributions of *G*. *morsitans centralis* and *G*. *swynnerton* (map drawn up based on data on each species in Rogers and Robinson 2004) [16].

*G*. *swynnertoni* is restricted to north-west Tanzania and south west Kenya [14,15], whereas *G*. *m*. *centralis* has a much more extensive distribution extending across the southern tsetse belt from western Tanzania and southern Uganda westwards through Zambia across the south-east of the Democratic Republic of the Congo to the eastern limits of Angola, with an isolated pocket in central Angola [16] (Fig 1). The former population in northern Botswana centred on the Okavango Delta, an important pastoral and conservation region, has been successfully eradicated following a concerted control programme of aerial spraying with insecticides and use of insecticide-impregnated visual targets in 2001 and 2002 [17]. The region has been tsetse-free for over 10 years [18].

Both *G*. *swynnertoni* and *G*. *m*. *centralis* are abundant in and around conservation areas [19] which are an important source of revenue particularly in Kenya, Tanzania [20] and parts of Zambia [21], but where effective vector management can be a particular challenge as an abundant wildlife reservoir means tsetse populations can reach high densities [22]. The transmission risk to neighbouring pastoralists and their livestock is very high, notwithstanding concerns for tourists, park staff [23], and even conservation programmes for endangered species such as the black rhinoceros *Diceros bicornis* [24,25].

Visually-attractive control devices such as insecticide-impregnated traps [26] and targets [27, 28] have been widely used to control savannah tsetse since the 1980s [29], including *G*. *m*. *centralis* [30] and *G*. *swynnertoni* [31, 32], although their use has been sporadic and often on a small scale [32]. The deployment of insecticide-impregnated targets alone has been successful in eliminating tsetse from geographically isolated pockets, such as the Lambwe Valley in Kenya [33]. They are a suitable environmentally friendly technique to use in joint efforts in and around game reserves [31] and have also been widely used to create barriers to prevent tsetse re-invading cleared areas [17, 30].

Large targets, up to 1.5–1.8 m wide, have been traditionally used in eastern and southern Africa to manage savannah tsetse populations [33, 34]. Some research has advocated the use of all-black targets for use against savannah tsetse [28, 35], but blue-black or all blue targets have been shown to be most effective against *Morsitans* group tsetse [36–38] and are now the most commonly advocated [34, 39].

Very large numbers of insecticide-impregnated targets need to be deployed and maintained to clear an area of tsetse and to create effective-barriers to prevent re-invasion. In the Tanzanian National Parks alone, over 20,000 targets were deployed between 2007 and 2010 [19]. The cost of materials, deployment and maintenance are major outlays and the traditionally large targets are also prone to wind damage in sandy soils and theft can also be a problem [30].

Recent research on riverine or *Palpalis* group tsetse has shown that much smaller targets (0.25–0.5 m^2^) can capture more flies per m^2^ than larger targets and would be more cost-effective in programmes targeting species such as *Glossina fuscipes fuscipes* and *G*. *palpalis palp*alis [40–43]. In contrast, for savannah species such as *G*. *morsitans morsitans* and *G*. *pallidipes*, research in Zimbabwe has shown that they would not be as effective as larger targets [28].

However, it appears that G. *swynnertoni* may respond differently to other savannah tsetse. Field trials by Mramba *et al*. [44] in the Serengeti made between 2009 and 2012, which were part of a pan-African WHO-TDR initiative on maximising the efficiency of visual baits for tsetse, showed that smaller sized horizontal (wider than high) 0.47 m^2^ blue leg panels and 0.5 m^2^ horizontal blue and blue-black-blue targets are equally efficient at inducing landing by *G*. *swynnertoni* as 1.5 m^2^ and 1 m^2^ blue-black or blue-black-blue targets previously used in East Africa. In these trials targets of 0.25 m^2^ were less efficient. Following on from these trials, we set out to identify the most appropriate reduced target shape and design for use as a visual control device for *G*. *swynnertoni*. Our aim was to maximise the efficiency and cost-effectiveness of these devices. The trials were repeated with G. *m*. *centralis*, a close relative of *G*. *swynnertoni*, to see whether this savannah species shared the same behavioural responses or was more akin to *G*. *m*. *morsitans*. Information on *G*. *pallidipes* is reported here where this species was also present at field sites.

Additional trials were also conducted with *G*. *m*. *centralis* to measure the performance of pyramidal and epsilon traps, which are still widely used to control and monitor this species. The relative performance of these traps was compared to targets with and without a chemical bait for *G*. *m*. *centralis*. Such information had already been collected for *G*. *swynnertoni* in an earlier set of trials [44].

## Materials and methods

### Study sites

Studies on *G*. *swynnertoni* and *G*. *pallidipes* were conducted at one site in 2013 in Tanzania. Studies on *G*. *m*. *centralis* were undertaken at two sites in central Angola (at one site in 2010 and at another in 2014) and at one site in the Democratic Republic of the Congo in 2014. A brief description of each site is given below. The field trials were made either on public land or on lands where owners/residents gave permission for the field trials to be conducted.

#### Angola, dry season, June 2014

The trials were carried out along 3 km of road, just outside Cabezo village [S 10º 16' 01'' E 15º 20' 01'' (altitude: 1178 m)] (200 inhabitants) in Cuanza Sul province, 525 km south-east of Luanda. The area is predominantly savannah grassland with small pockets of farmland, used mainly for growing manioc. The main livestock are goats and chickens and wild animals, mainly bush pigs and antelopes, are common.

#### Angola, wet season, May 2010

The trials were undertaken in savannah grassland/wooded savannah near the village of Quissongo, Calulo in Cuanza-Sul province [S 10° 06’ 29” E 15° 10’ 36”] (altitude: 980 m). There were no livestock (except poultry) but the population of wild animals (monkeys, antelopes) was relatively high.

#### Democratic republic of the congo (DRC), wet season, March 2014

The trial site was situated near the villages of Kisima and Kalamba near the confluence of the Luizi and Luyeye rivers (tributaries of the Lukuga), near Nyunzu, 194 km west of Kalemie in the south-east of the Democratic Republic of the Congo [S 6°04’ 30”/ E 27°39’ 14”; (altitude: 640 m)]. The natural vegetation of the region is open wooded savannah (Miombo type) dominated by *Panicum* spp., *Hyparrhenia displandra*, *Acacia* spp. and palms in a mosaic with numerous swamps and fens. It is an area of intensive human activity (farming, fishing and hunting) with significant movements of domestic animals, notably sheep, goats, pigs and hunting dogs. Indigenous fauna includes wild boars, hares and several species of antelope.

#### Tanzania, end of the wet season, July 2013

Open savannah woodland (*Acacia—Commiphora*) in Death Valley near Seronera Lodge in the Serengeti National Park, Tanzania [S 2° 22’ 21” E 34° 43’ 08”, (altitude: 1548 m)]. Wildlife (wildebeest, buffalo, zebra, gazelles) is abundant in the area but there are no domestic livestock. The population density of *G*. *pallidipes* was much lower than that of *G*. *swynnertoni* at the site as already recorded in experiments between 2009 and 2012 [44].

### Visual devices and materials

In all three countries a series of 1 m^2^ and 0.5 m^2^ rectangular and square targets made of equal vertical rectangles of blue and black or all-blue cloth were tested (Table 1). Rectangular targets, termed here horizontal oblongs, had their long sides set up horizontal to the ground. A selection of different dimensions and designs was chosen to assess the influence of target shape, size and colour on fly landing rates. In addition, pyramidal traps [45] were included in the 2014 Angolan and Congolese trials and epsilon traps [46] were used in the 2010 Angolan trials. Catches and landing rates for pyramidal traps were compared with landing rates on targets in trials carried out earlier at the same site in Tanzania [44] and so were not repeated. All devices were set in the open, 30 cm above ground, and vegetation was removed within several metres of each device.

**Table 1:** Trapping devices used and their surface areas.

Object	Type and colour combination#	Size	Sticky surface area
**Tanzania 2013**			
1 m^2^ square target	blue/black 1:1	1 x 1 m	2 m^2^
0.5 m^2^ horizontal oblong	blue/black 1:1	0.9 x 0.55 m	1 m^2^
	all-blue	0.9 x 0.55 m	1 m^2^
0.5 m^2^ square target	blue/black 1:1	0.71 x 0.71 m	1 m^2^
	all-blue	0.71 x 0.71 m	1 m^2^
**Angola and DR Congo 2014**			
Pyramidal trap	blue/black 1:1		2 m^2^
1 m^2^ horizontal oblong	blue/black 1:1	1.25 x 0.8 m	1 m^2^
0.5 m^2^ horizontal oblong	blue/black 1:1	0.9 x 0.55 m	1 m^2^
	all-blue	0.9 x 0.55 m	1 m^2^
0.5 m^2^ square target	blue/black 1:1	0.71 x 0.71 m	1 m^2^
**Angola 2010**			
Pyramidal trap	blue/black 1:1		N/A
Epsilon trap	all-blue		N/A
1 m^2^ square target	blue/black 1:1	1 x 1 m	2 m^2^
1 m^2^ square adhesive film	N/A	1 x 1 m	1 m^2^

# Blue/black targets are divided vertically into equal parts of blue and black cloth.

In all devices except the epsilon trap, two fabrics were used: C180 Azur 623 phthalogen blue 100% cotton (180 g/m^2^, TDV, Laval, France) with a reflectance peak at 460 nm as measured with a Datacolor Check Spectrophotometer (Datacolor AG, Dietlikon, Switzerland) and a 100% polyester black (225 g/m^2^, Q15093 Sunflag, Nairobi). The epsilon trap was made of blue polyester (PermaNet, Vestergaard Fransen, Denmark), also with a reflectance peak at 460 nm (see Supporting Information S1 Fig for spectral reflectance curves).

To enumerate flies landing on the devices, one-sided adhesive film (30 cm wide rolls, Rentokil FE45, Liverpool, UK) was stitched onto both sides of the targets and onto the cloth panels of the pyramidal traps. These fly catches permitted measurement of tsetse landing rates on the different devices, the essential behavioural response underlying the use of insecticide-impregnated visual control devices for tsetse. The adhesive film does not affect spectral reflectance except in the ultra-violet spectrum, absorbing virtually all UV wavelengths below 380 nm. This is due to the addition of a UV absorber in the glue. In addition, spectrophotometric measurements of light reflected from adhesive film applied onto the same fabrics used in this study indicate that all wavelengths in the UV range were mostly absorbed by the fabrics [43]. In the 2010 Angolan trial, a 1 x 1 m square of adhesive film alone (without any cloth backing) was compared to cloth targets with adhesive film attached to both sides to ascertain whether adhesive film in itself attracts flies.

A 1:4:8 mixture of 3-n-propylphenol (P), 1-octen-3-ol (O), and p-cresol (C) (Ubichem Research LTD, Budapest/Hungary, global purity of 98%) with acetone (A) was used as an attractant for experiments comparing performance ranking of baited devices based on its efficacy for several tsetse species. This combination is termed POCA bait and was made up as per Torr *et al*. [47]. Sachets of 4 cm x 5 cm 500 gauge / 0.125 mm polyethylene containing 3 g of the 1:4:8 mixture were placed below the visual devices, 10 cm above ground, alongside a 250 ml bottle buried up to the shoulders containing acetone with a 2 mm aperture in the stopper.

### Experimental design

#### Evaluating the best landing device

These experiments were carried out to evaluate the efficacy of the different unbaited targets at inducing fly landings. Traps with and without adhesive film were included in Angola and the DRC to estimate the landing and trapping efficiency of the pyramidal device for *G*. *m*. *centralis*. Five to six devices were compared in each trial in Latin square design experiments of days x sites x treatments, with three simultaneous replicates. The trapping positions were always > 100 m apart and flies of each sex from each device were counted after 24 hours at each position.

#### Evaluating the influence of POCA bait on trap entry

Trials of devices with and without the POCA bait were made in Angola to determine whether baiting increased the proportion of flies entering the cages of traps relative to the number landing on targets. The performance of the epsilon and pyramidal traps was compared with a 1 m^2^ square target with equal vertical rectangles of blue and black and with a target made of 1 m^2^ of adhesive film. Experiments followed a Latin square design of days x sites x treatments, with four simultaneous replicates. Baited trapping positions were > 200 m apart because the attractants can be effective up to 100 m downwind. To avoid contamination of devices with odours from baits, the trial without baits was made first and repeated shortly after with POCA-baited devices in the same general area. Consequently, only the relative performance of devices within a trial is interpreted and not counts of flies on devices between these two consecutive experiments.

### Statistical analysis

In all field trials randomization was set up using design.lsd in the package agricolae [48] R version 3.01 [49]. Data were analysed using a linear model including the following additional packages: MASS [50] and multcomp [51]. Analysis was performed on log (x+1) transformed data including day and position as additional explanatory parameters. Position had no significant effect in any field trial (P > 0.05, F-test) and running the model separately for replicates also revealed no significant effect in any of the field trials (P > 0.3, F-test). Tukey contrasts were calculated to compare treatments. The Wilcoxon paired test was used to compare fly landings on the blue and black portions of targets.

## Results

### Best landing devices

**Fig 2:**
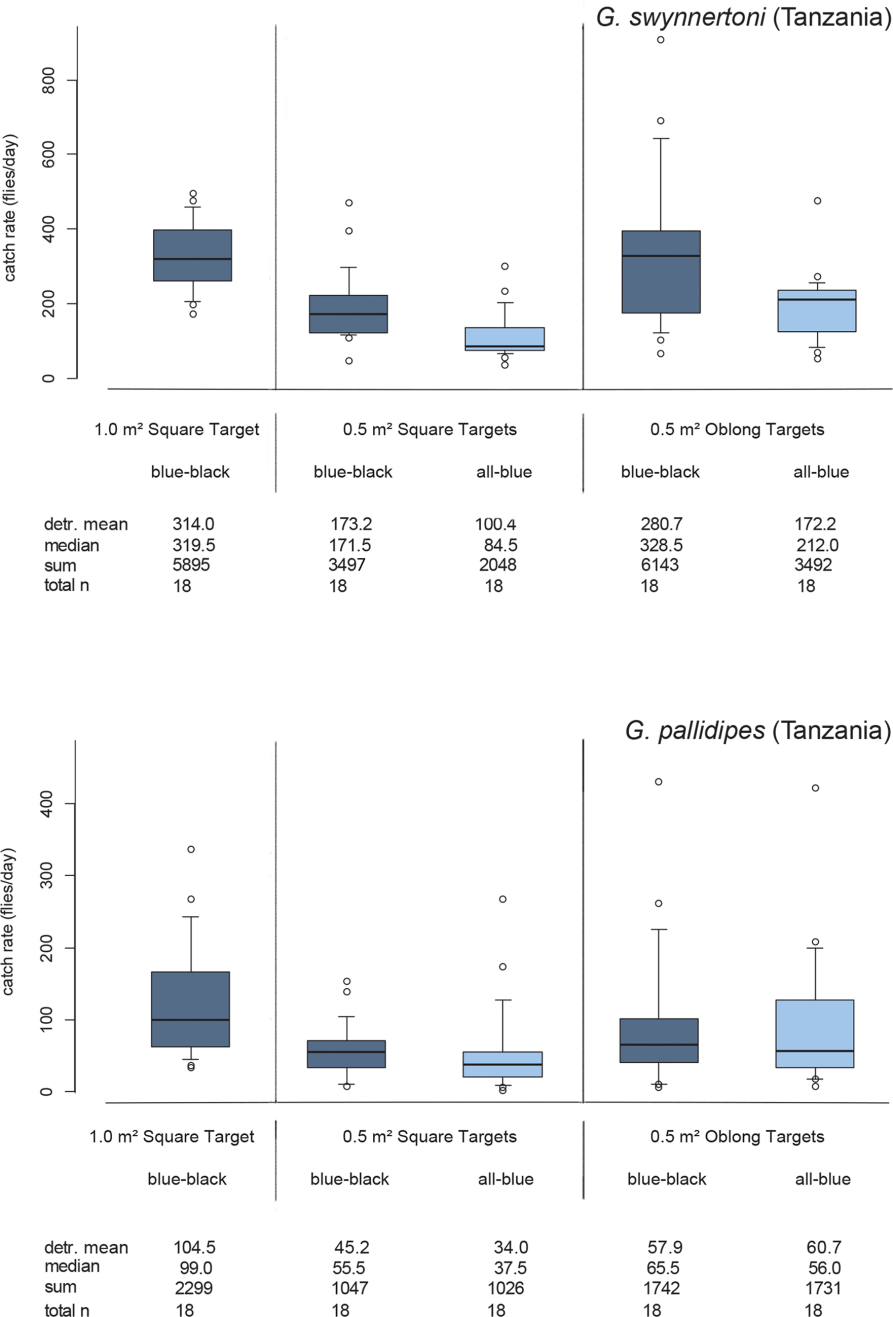
Daily catch rates of *G*. *swynnertoni* and *G*. *pallidipes* in Tanzania on different visual targets. The limits of the boxes indicate the twenty-fifth and seventy-fifth percentiles, the solid line in the box is the median, the capped bars indicate the tenth and the ninetieth percentiles, and data points outside these limits are plotted as circles; dtr. mean is the detransformed mean.

**Fig 3:**
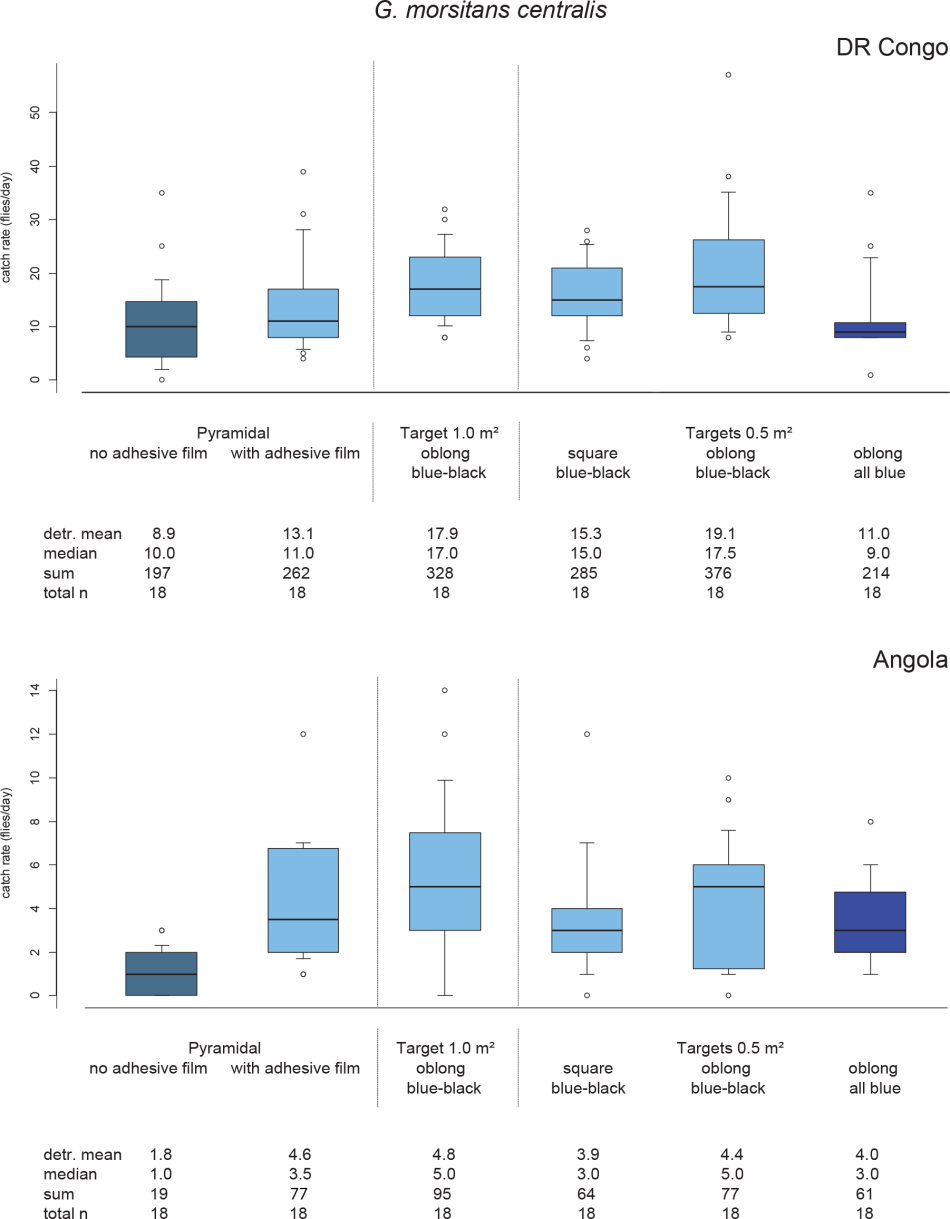
Daily catch rates for *G*. *m*. *centralis* by different visual devices in Angola and DR Congo. The limits of the boxes indicate the twenty-fifth and seventy-fifth percentiles, the solid line in the box is the median, the capped bars indicate the tenth and the ninetieth percentiles, and data points outside these limits are plotted as circles; dtr. mean is the detransformed mean.

The largest target tested induced the highest number of *G*. *swynnertoni* and *G*. *pallidipes* to land on it, but this was not significantly greater than the daily landing rates for the 0.5 m^2^ blue-black oblong target for both species (P > 0.05; Table 2 & Fig 2). Landings on the 0.5 m^2^ blue/black oblong were particularly high for *G*. *swynnertoni* (90% of the daily landing rate on the 1 m^2^ square target). For *G*. *m*. *centralis*, daily landing rates were highest on the 1 and 0.5 m^2^ blue-black oblong targets but were not significantly different (P > 0.05) from the sticky pyramidal trap and 0.5 m^2^ square blue-black target (Table 2 & Fig 3).

**Table 2:** Daily landing rates and catches of *G*. *swynnertoni*, *G*. *m*. *centralis* and *G*. *pallidipes*, respectively, on different targets and in pyramidal traps.

Device	colour	*G*. *swynnertoni*	*G*. *pallidipes*	*G*.* m*.* centralis*	
		*Tanzania*		*DR Congo*	*Angola*
1 m^2^ square target	blue/black	314.0 ^a^	104.5 ^a^		
1 m^2^ horizontal oblong	blue/black			17.9 ^a^	4.8 ^b^
0.5 m^2^ horizontal oblong	blue/black	280.7 ^ab^	57.9 ^ab^	19.1 ^a^	4.4 ^b^
	all-blue	172.2 ^b^	60.7 ^ab^	11.0 ^bc^	4.0 ^b^
0.5 m^2^ square target	blue/black	173.2 ^b^	45.2 ^b^	15.3 ^ab^	3.9 ^b^
	all-blue	100.4 ^c^	34.0 ^b^		
Pyramidal trap	blue/black			8.9 ^c^	1.8 ^a^
Pyramidal trap with adhesive film	blue/black			13.1 ^abc^	4.6 ^b^

In each column, detransformed means followed by a different letter are significantly different (Tukey post hoc test, P < 0.05).

See also Supporting Information S1 Table for detransformed mean daily landing rates and catches (with transformed means ± standard errors in brackets, natural logarithms).

### Optimal target size, shape, colour & trap efficiency

The largest square blue-black targets (1 m^2^) showed the highest landing rates for *G*. *swynnertoni* and *G*. *pallidipes;* landings were reduced by around 50% on the equivalent 0.5 m^2^ square targets (Table 2 & Fig 2). For both species, landing rates were higher on the 0.5 m^2^ blue-black oblong targets than on the equivalent square targets, most noticeably for *G*. *swynnertoni* (62% more) but this difference was not significant (P > 0.05).

The daily landing rates for *G*. *m*. *centralis* in the DRC were nearly the same on the 1 and 0.5 m^2^ blue-black oblong targets (17.9 and 19.1 flies per day, respectively), which were 20–25% more than on the equivalent 0.5 m^2^ square target (Table 2 & Fig 3). Very similar trends were observed in the smaller landing rates recorded in Angola. None of these differences were significant (P > 0.05).

The blue-black targets performed better than their equivalents in all-blue for both *G*. *swynnertoni* and *G*. *m*. *centralis*, with landings significantly lower for *G*. *swynnertoni* on the all-blue square target and for *G*. *m*. *centralis* in the DRC on the all-blue oblong target (P < 0.05). In contrast, there was no significant difference between daily landing rates for *G*. *pallidipes* on the blue-black and equivalent all-blue 0.5 m^2^ oblong and square targets (Table 2). Landing rates relative to colour / size / shape were also equivalent for the experiment conducted in Angola at low numbers of *G*. *m*. *centralis*.

When the daily landing rates are adjusted to a uniform size of 1 m^2^ for the targets of various shapes and sizes, the optimal landing rates were recorded on the 0.5 m^2^ blue-black oblong targets for all three species (Table 3). *Glossina pallidipes* was the only species with similar landing rates on the 0.5 m^2^ all-blue oblong (Table 3). Although landing rates per m^2^ were approximately double those of the 1 m^2^ targets for *G*. *swynnertoni* and *G*. *m*. *centralis*, landing rates per m^2^ were only slightly higher (~10%) for *G*. *pallidipes*, and were actually lower for the same shaped smaller target.

**Table 3:** Tsetse landing indices (detransformed mean daily landings) per m^2^ and per m edge of different sized targets.

	Target			Mean daily landing rates			
	size	shape	colour	whole target	flies per m^2^	flies per m edge	flies per m horizontal edge
***G*.* swynnertoni***	1 m^2^	square	blue/black	**314**	157.0	78.5	157.0
	1 m^2^	oblong	blue/black				
	0.5 m^2^	square	blue/black	173	173.0	60.7	123.6
	0.5 m^2^	oblong	blue/black	281.0	**281.0**	96.9	156.1
	0.5 m^2^	square	all-blue	100.4	100.4	35.1	71.4
	0.5 m^2^	oblong	all-blue	172.2	172.2	59.3	95.6
***G. m*.* centralis* (DRC)**	1 m^2^	square	blue/black				
	1 m^2^	oblong	blue/black	17.9	9.0	4.4	7.2
	0.5 m^2^	square	blue/black	15.3	15.3	5.3	10.7
	0.5 m^2^	oblong	blue/black	**19.1**	**19.1**	6.6	10.6
	0.5 m^2^	square	all-blue				
	0.5 m^2^	oblong	all-blue	11.0	11.0	3.8	6.1
***G*.* m*.* centralis* (Angola)**	1 m^2^	square	blue/black				
	1 m^2^	oblong	blue/black	**4.8**	2.4	1.2	1.9
	0.5 m^2^	square	blue/black	3.9	3.9	1.4	2.7
	0.5 m^2^	oblong	blue/black	4.4	**4.4**	1.5	2.4
	0.5 m^2^	square	all-blue				
	0.5 m^2^	oblong	all-blue	4.0	4.0	1.4	2.2
***G*.* pallidipes***	1 m^2^	square	blue/black	**104.5**	52.5	26.3	52.5
	1 m^2^	oblong	blue/black				
	0.5 m^2^	square	blue/black	45.2	45.2	15.8	32.1
	0.5 m^2^	oblong	blue/black	57.9	57.9	20.0	32.2
	0.5 m^2^	square	all-blue	34.0	34.0	11.9	24.3
	0.5 m^2^	oblong	all-blue	60.7	**60.7**	20.7	33.3

#### Target horizontal edge effects

Daily landing rates normalised to per m edge of target did not show a strong correlation between the number of flies landing and the length of target edge. However, when the landing rates are normalised to an equal length of horizontal edge there was a closer correlation with number of flies landing for all three species, particularly for *G*. *m*. *centralis* and *G*. *pallidipes* on the 0.5 m^2^ blue-black targets (Table 3).

#### Tsetse colour preferences on traps and targets

All three species showed a strong preference for landing on the blue portions of the trap and targets, with little difference between devices and sexes (Supporting Information S2 Table). The blue landing bias was pronounced in *G*. *swynnertoni* and *G*. *pallidipes*, with at least three times as many flies landing on blue compared to black. For *G*. *m*. *centralis*, roughly 1.5–2 times as many flies landed on blue compared to black. For all species, the blue preference was significant (P < 0.05, Wilcoxon Test). However, the application of adhesive film can reduce landings on black materials in other tsetse species (see Discussion).

#### Trapping efficiency and efficiency of traps as control devices

Trap efficiency is defined as fly numbers caught in the cage as a proportion of the total number landing on/entering the trap. As in Mramba et al. [44] it was estimated by dividing the mean daily catch in cages of unaltered pyramidal traps by the mean daily catch of the matching traps with adhesive film on the cloth (flies caught on the adhesive film and in the cage; see Fig 3). This definition is conceptually different from studies working with e-nets, which are based on the interception of circling flies attracted to the vicinity of the device [43]. From these results, pyramidal trap efficiency for *G*. *m*. *centralis* was estimated at 25% in Angola and 68% in the Democratic Republic of the Congo. In this study we were interested in comparing the killing efficiency, i.e. actual numbers landing on trap panels, to evaluate them as control devices. The relative efficiency of the pyramidal traps as control devices was estimated by dividing the mean daily landing rate on the trap with adhesive film on its cloth panels by the mean daily landing rate on the best performing target. From these results the landing efficiency of the pyramidal trap compared to the target is 70–80% for *G*. *m*. *centralis*.

### Influence of POCA bait on trap entry

The rank order in performance of devices was the same in the baited and unabaited experiments (Table 4). Addition of POCA bait had no influence on the proportion of *G*. *m*. *centralis* flies entering the epsilon trap relative to landings on the blue-black cloth target, with only slightly fewer flies entering the baited trap compared to that recorded in the unbaited experiment (Table 4). In contrast, the addition of the POCA bait increased the proportion of flies entering the pyramidal trap compared to landings on the cloth target by over 60% (Table 4).

**Table 4:** Daily landing rates and catches of *G*. *m*. *centralis*, respectively, on targets and in different trap types with and without the POCA bait.

	Device	Colour	*G*.* m*.* centralis*	
		*Unbaited*	*POCA-baited*
Pyramidal trap	blue/black	11.1^a^	27.1^b^
Epsilon trap	all-blue	8.8^a^	10.7^a^
1 m^2^ square target with adhesive film	blue/black	52.4^b^	78.8^c^
1 m^2^ square adhesive film	clear	6.1^a^	8.8^a^

In each column, detransformed means followed by a different letter are significantly different (Tukey post hoc test, P < 0.05).

See also Supporting Information S3 Table for detransformed mean daily landing rates and catches (with transformed means ± standard errors in brackets, natural logarithms).

## Discussion

### Performance of traps versus targets as landing devices

One of the objectives of the present study was to quantify the performance of pyramidal traps relative to targets for several savannah tsetse, as this trap (or similar monoconical traps such as the Vavoua) is often used as a generic tsetse sampling device in areas with many species [42,43,44,52]. In Tanzania, pyramidal traps are often used for sampling *G*. *swynnertoni* populations (following the early work of Muangirwa [53]), but landing efficiency is about 50% lower than for a target, and trapping efficiency only about a quarter [44]. Despite this, pyramidal traps continue to be used for monitoring for practical reasons, although their large-scale use in the control of *G*. *swynnertoni* is not recommended. We have no explanation for the difference in trapping efficiency of the pyramidal trap for *G*. *m*. *centralis* estimated at 25% in Angola and 68% in the DRC other than to note that the pertinent field trial was made in the dry season in Angola and in the wet season in the DRC.

**Fig 4:**
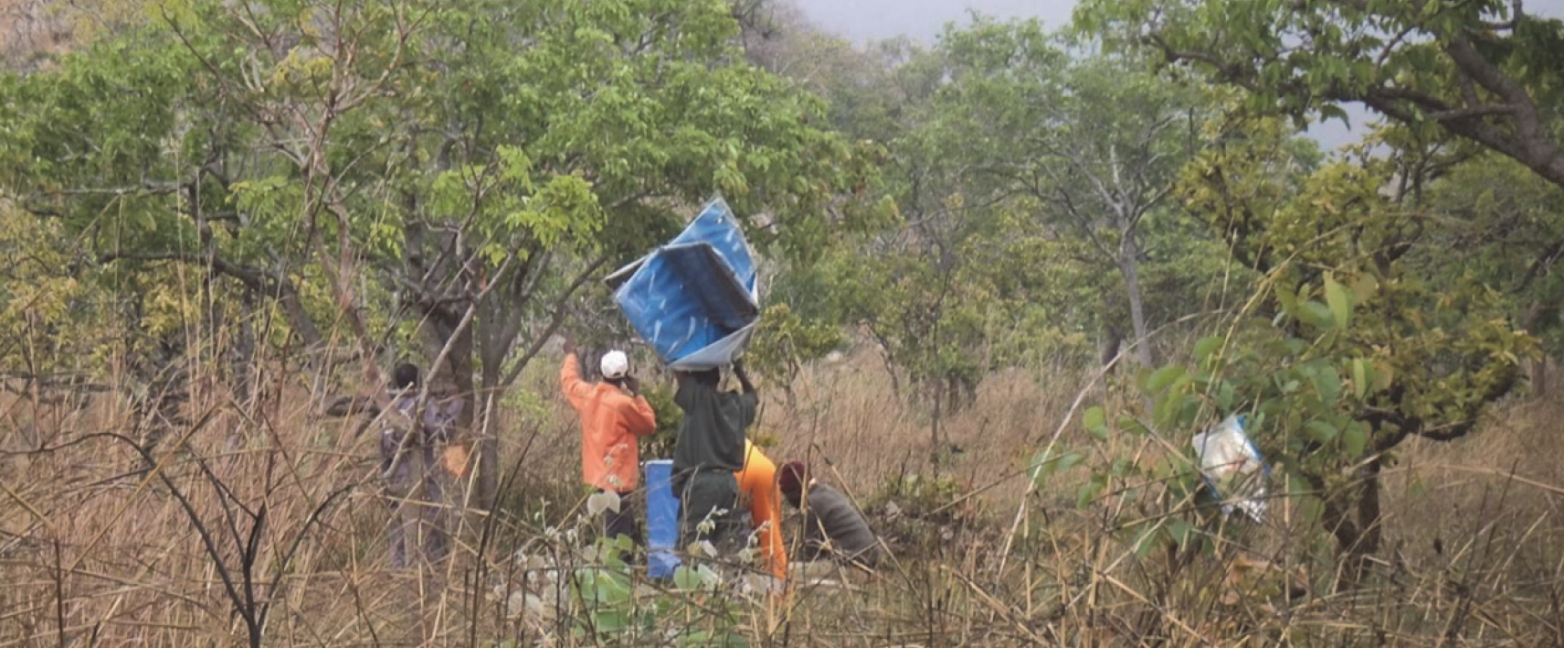
Suspending a pyramidal trap with adhesive film on its panels from a tree in wooded savannah, Cabezo, central Angola.

In Angola, where *G*. *m*. *centralis* is present, insecticide-impregnated pyramidal traps rather than targets are widely-used for tsetse control [cf. 42]. Here, we show that the number of *G*. *m*. *centralis* landing on pyramidal traps covered with adhesive film is similar to, but somewhat lower than, the numbers landing on the best blue-black target (68–95% of the best target in two experiments). Also, no flies were captured in the cages of the sticky traps in these trials. This tsetse species [54], like most savannah species [44,52], seems to have a very low propensity to enter a trap cage without first landing on the cloth, unlike some riverine species such as *G*. *palpalis palpalis* [42]. This behavioural trait combined with the relative attractiveness of the pyramidal trap means that insecticide-impregnated pyramidal traps are sufficiently effective fly-killing devices to support their continued deployment for the control of *G*. *m*. *centralis* [42]. In countries such as Angola, hanging traps from bushes and stems of trees is a typical deployment strategy in wooded savannah (Fig 4) where it has proven to be more practical and economical than implanting supports for targets in the ground [55].

### Optimal target shape, configuration and colour

This current study was carried out following on from other target/trap comparisons we have made across Africa [42,43,44,52], and hence our trials focused on optimizing lessons learned in previous work, particularly the unexpected finding of the high performance of small targets for savannah species in Tanzania and Kenya [44]. In our current trials in three countries for three related savannah species, the highest landing rates were most frequently recorded on the 1 m^2^ blue-black target that we adopted as a standard for coordinated experiments. This design was expected to be highly-attractive, even when unbaited, based on a large body of previous work by many researchers [34]. However, our results, consistent with Mramba et al.’s earlier findings on *G*. *swynnertoni* [44], also show that some smaller 0.5 m^2^ targets can be just as efficient for other savannah species (highest efficiency index in terms of fly landings per m^2^ of cloth with statistically equivalent total landings relative to a 1 m^2^ blue-black target) and should be considered as sampling/control devices.

These consistent findings for several species in different countries contrast with results of a test of a “tiny” target (0.06 m^2^) for two savannah species in Zimbabwe (*G*. *pallidipes* and *G*. *m*. *morsitans*), where Torr et al. [28] found that very few tsetse were attracted to or landed on a 0.25 x 0.25 m square, all-black target (with and without flanking e-nets and/or baits). The simplest explanation for such dramatically different results among experiments in different countries (given that *G*. *pallidipes* is represented in both sets of trials) is that the Zimbabwe trials tested only small targets that were all-black, i.e. without a blue element. In the key studies leading up to modern targets for savannah tsetse, Vale [56] concluded that bicoloured blue/black panels would make the best targets, and there are many examples of the importance of blue in tsetse vision [57,58]. The presence of a contrasting blue element may be critical for attracting certain tsetse to small versus large targets [40].

Any interpretation of the importance of blue cannot necessarily be inferred from the preferential landing by tsetse on the blue portions of devices tested here (Supporting Information S2 Table). In earlier studies designed to assess the effect of adding adhesive film to visual devices, blue-black 1 m^2^ targets with no adhesive film applied, and similar targets covered on both sides by adhesive film with the sticky side facing inwards (i.e. with the shiny plastic base facing outwards), were placed within electric grids designed to kill alighting flies [42,52]. These experiments showed that addition of a specular component to the light reflecting from cloth significantly reduced landings on the black but not the blue portion of targets for other tsetse species (*G*. *p*. *palpalis*, *G*. *tachinoides* and *G*. *gambiensis*). As the Rentokil film is also selectively UV-absorbing, these effects could have been due to the fact that the appearance of matt-finished phthalogen blue cotton and black polyester fabrics was also altered in terms of UV reflectance. High UV reflectance is typically assumed to negatively affect tsetse responses to objects independent of visible reflectance based on statistical trends in tests of a wide variety of materials. However, spectrophotometric measurements of light reflected from adhesive film applied onto the phthalogen blue cotton and black polyester fabrics as on tsetse visual devices indicate that all wavelengths in the UV range were in any case mostly absorbed by the fabrics [43]. Also, the adhesive film served to increase landings by *G*. *palpalis gambiensis* on the blue portion of targets [52]. This suggests that other complex fly visual phenomena may be at play [59] and serves to underline that colour preferences using this sticky method of enumeration should be interpreted with caution [42,52]. Also, as noted by Vale [56], blue-black targets generally perform better for savannah species, including *G*. *pallidipes* and *G*. *m*. *morsitans*, than all-black targets [36,38]. As a relevant example, Knols et al. [25] gradually replaced 1.8 m wide x 1 m tall all-black targets of the Zimbabwe design with bicoloured blue-black targets for the control of *G*. *m*. *centralis* in Zambia. Lastly, the presence of a blue element of the correct spectral characteristics (including ultraviolet reflectance [58]) has been shown to be important in the optimization of small targets for riverine tsetse [40,43,60].

The use of very small targets (i.e. 0.25 m^2^ or smaller) as proposed for some riverine tsetse [40,41] may not prove to be suitable for savannah tsetse, given our previous results for 0.25 m^2^ targets for *G*. *swynnertoni* [44], and the poor results for all-black “tiny” targets from Zimbabwe cited above [28]. Nevertheless, the cost benefit and other practical implications of deploying targets somewhat smaller than 1–1.5 m^2^ warrant serious consideration. Control campaigns and the establishment of barriers against re-invasion require thousands to tens of thousands of visual targets, hence “size matters” [17,31]. In addition, in regions where wind damage and implanting supports for targets can be difficult (very hard ground or loose sandy soils [30]) deploying smaller targets is a practical solution, provided they remain efficient at inducing landing when left *in situ* for long periods of time. We therefore continue below with a more focused discussion of the performance of the 0.5 m^2^ target designs for the three savannah species studied here.

### Influence of target configuration: Blue-black versus all-blue

In Tanzania, equal vertical rectangles of blue-black-blue have been traditionally used as targets for tsetse following the initial recommendations of Vale [56] in Zimbabwe. However, as we previously found no difference in the performance of blue-black-blue and blue-black targets in phthalogen blue cloth for inducing landing by *G*. *swynnertoni* [44], we used the simpler blue-black configuration for further tests of smaller targets. For *G*. *swynnertoni*, and *G*. *m*. *centralis* (in the DRC), landings on the blue-black targets were 55–75% higher than on the all-blue devices. A black portion was therefore an essential element for inducing landing in these two species and its contribution would probably have been more significant in absence of the adhesive film (see above). This is in contrast to *G*. *pallidipes*, where all-blue targets were found to be as efficient as blue-black targets. *Glossina m*. *centralis* is genetically closer to *G*. *swynnertoni* than to *G*. *m*. *morsitans* [6] and this may be a reason why its landing behaviour is more similar to the former. The higher preference for the blue-black over the all-blue devices by *G*. *swynnertoni* is greater than the 30% increase recorded earlier by Mramba et al. [44] at the same site, when flies were more evenly distributed between blue and black. This may be a seasonal difference as revealed in the early work of Vale [56].

### Influence of target shape: Square versus horizontal oblong

Horizontal oblong targets appear to be better at inducing landing than square targets for certain tsetse species, such as the riverine species *G*. *tachinoides* [43,61] and *G*. *f*. *fuscipes* [40,43], especially for smaller targets. Increasing target width has also been found to increase landing rates by certain savannah species, such as *G*. *austeni* [62] and *G*. *m*. *morsitans* and *G*. *pallidipes* [55]. We therefore tested whether shape was a factor affecting landing efficiency for the three savannah tsetse species studied here using carefully-matched small target designs. We found that in all countries, irrespective of savannah species or season, horizontal oblong targets were better at inducing landing than an equivalent size square target, confirming our initial supposition.

### Target horizontal edge effects on fly landing

Our results have shown a close predictive correlation between the length of horizontal edge and tsetse landing rates independent of colour for all three species, in the target size ranges investigated. The exploitation of edge or border effects through the incorporation of simple geometric shapes/borders is a relatively unexplored area of research for improving targets for tsetse [63] as is the colour/background contrasts in targets [64]. In the extensive literature on tsetse visual responses, only a few researchers have systematically examined how tsetse land on different parts of large targets in relation to potential edge and colour/contrast effects [56, 61]. Since Vale established that most tsetse land in the centre of targets [56] it could be that visual targets with a longer horizontal aspect with respect to ground are better at accommodating landings by fast-flying tsetse. In the laboratory, a preference for the edges of objects by *G*. *m*. *morsitans* is a particularly interesting finding [65]. A similar landing preference for the horizontal edge of targets by *G*. *f*. *fuscipes* was observed in the field by Oloo et al [43]. Vreysen et al [61] tested targets with horizontal or diagonal arrangements of solid blocks of colour to discern the impact on total landings by *G*. *austeni* and found a strong preference for the bottom corner edge. If the three species studied here truly have similar innate behavioural responses to a horizontal edge phenomenon, this could explain why the horizontal oblong, with a higher edge/surface area ratio than the square, and with longer upper and lower edges than the square, was more efficient at inducing landings.

### POCA bait and trap entry

The use of the POCA bait has been shown to increase trap entry by flies for several savannah tsetse, notably *G*. *m*. *morsitans* and *G*. *pallidipes* [66]. Trials in Kenya and Tanzania on *G*. *swynnertoni* [44] showed that POCA could double pyramidal trap entry relative to landing on blue-black targets, but this increase was inconsistent and was not recorded in all circumstances.

In this study in Angola, in a single trial with *G*. *m*. *centralis*, the addition of POCA increased pyramidal trap entry by 60% compared to an unbaited trap, relative to landings on the target. In contrast the POCA bait did not influence on entry into the epsilon trap. Earlier work has already shown that the epsilon trap catches fewer *G*. *swynnertoni* than conical trap designs [53] and would appear unsuitable in these countries as a monitoring device for these two species. Its single entry point and the fact that it is placed lower on the ground where it is more easily hidden by tall grass may also be contributory factors. However, in contrast, the epsilon trap has proved to be a satisfactory tsetse trapping/monitoring device in southern Africa (e.g. in Botswana and Zimbabwe) [67]. Considering the very modest improvements in trap entry by *G*. *m*. *centralis* with POCA, which are similar to earlier results with *G*. *swynnertoni* [44], and previous failures to substantially improve *G*. *swynnertoni* catches with chemical baits (i.e. double or more) [54, 68,69], there appears to be little benefit in deploying and maintaining baits for controlling these species. Simply increasing the deployment of smaller targets may be a more cost-effective strategy.

### Concluding remarks

This study has confirmed earlier findings on *G*. *swynnertoni* that smaller visual targets, down to 0.5 m^2^, would be as efficient as using 1 m^2^ targets visual targets for this species. This is also the case for *G*. *m*. *centralis*. To maximise the efficiency of smaller targets, horizontal rectangles with respect to ground that have both a black and phthalogen blue element appear to be best. These two features induced the highest landing response. All-blue devices were as efficient as blue-black devices for *G*. *pallidipes*. Adhesive film was used as a convenient alternative to other techniques to catch flies landing on visual devices at the remote locations to compare tsetse-landing efficiencies. However, because of interpretation difficulties inherent to the use of adhesive film to catch flies that land on visual targets, further studies with other techniques for intercepting flies landing on or circling targets (e.g. electric grids and targets with netting panels) are nevertheless still needed to better define the most economical and practical target for the control of all three species.

Insecticide-impregnated pyramidal traps are also effective devices for the control of *G*. *m*. *centralis* as they induce a strong landing response and hence would achieve the desired end-point of killing flies. Although they are not as economical as smaller targets, their continued use would be appropriate where hanging traps from tree branches would be less problematic than the implantation of supports for targets in the ground (e.g. in wooded savannah).

## Supporting information

S1 Fig Spectral reflectance measurements for materials used in traps and targets. TDV C180 is pure cotton dyed with a genuine Phthalogen Blue dyestuff from Dystar, Germany which precipitates as copper phthalocyanine in the fabric (Pigment Blue 15 or C.I. 74160).
(TIF)

S1 Table Mean daily landing rates and catches of tsetse. Detransformed mean daily landing rates and catches (with transformed means ± standard errors in brackets, natural logarithms) of *G*. *swynnertoni*, *G*. *m*. *centralis* and *G*. *pallidipes*, respectively, on different visual targets and in pyramidal traps.
(DOCX)

S2 Table Landing rate distributions of three tsetse species. Landing rate distributions of *G*. *swynnertoni*, *G*. *m*. *centralis* and *G*. *pallidipes* according to sex and substrate colour on visual targets and the pyramidal trap.
(DOCX)

S3 Table Mean daily landing rates and catches of *G*. *m*. *centralis*. Detransformed mean daily landing rates and catches (with transformed means ± standard errors in brackets, natural logarithms) of *G*. *m*. *centralis* on a blue-black visual target, a clear target made of adhesive film, and in different traps, with and without the POCA bait.
(DOCX)
