# Supplementary figures and images for: Standardising visual control devices for Tsetse: East and Central African Savannah species Glossina swynnertoni, Glossina morsitans centralis and Glossina pallidipes
Source: PLoS Negl Trop Dis. 2018 Sep 25;12(9):e0006831. doi: 10.1371/journal.pntd.0006831 (PMC6173441; doi:10.1371/journal.pntd.0006831)

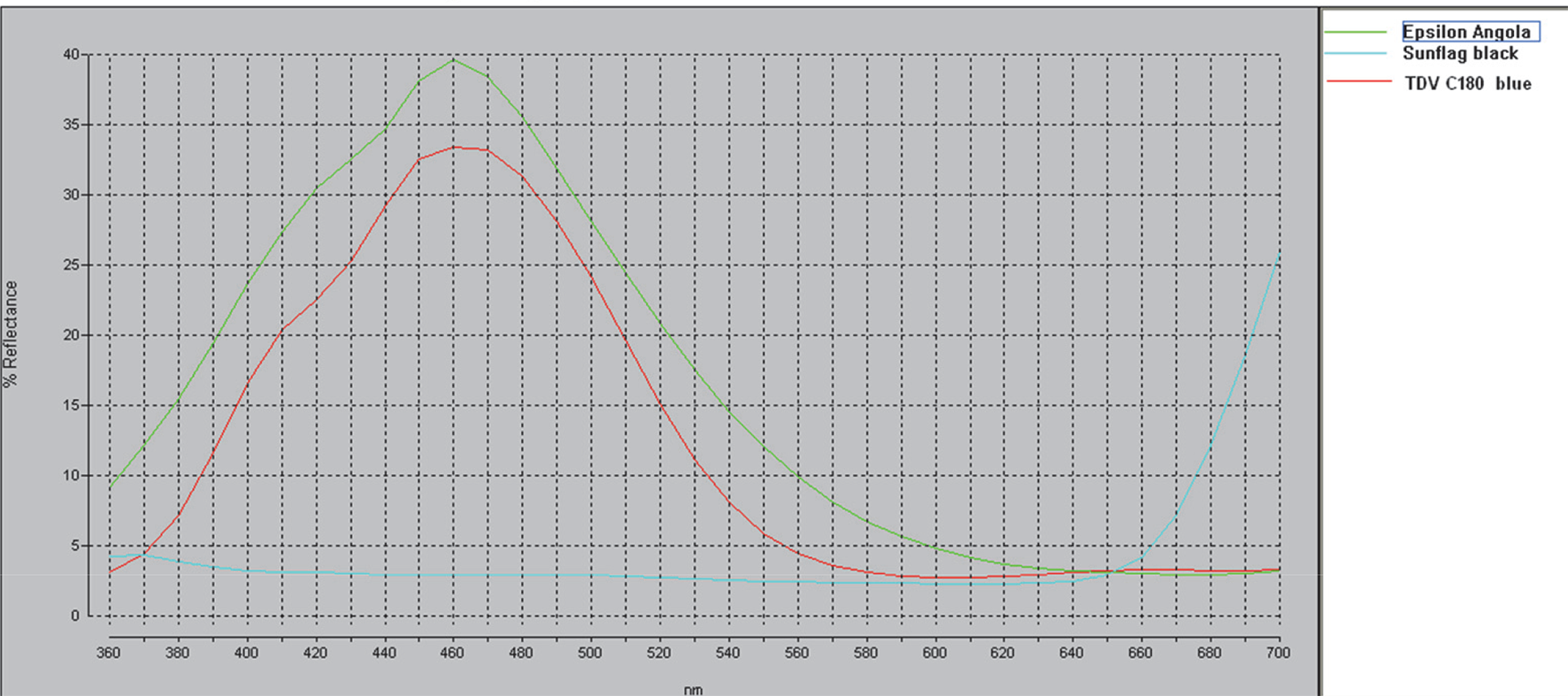

Supplement: S1 Fig — TDV C180 is pure cotton dyed with a genuine Phthalogen Blue dyestuff from Dystar, Germany which precipitates as copper phthalocyanine in the fabric (Pigment Blue 15 or C.I. 74160). (TIF) [file pntd.0006831.s001.tif]
