# Supplementary material for: Standardising visual control devices for Tsetse: East and Central African Savannah species Glossina swynnertoni, Glossina morsitans centralis and Glossina pallidipes
Source: PLoS Negl Trop Dis. 2018 Sep 25;12(9):e0006831. doi: 10.1371/journal.pntd.0006831 (PMC6173441; doi:10.1371/journal.pntd.0006831)
Supplement: S1 Table — Detransformed mean daily landing rates and catches (with transformed means ± standard errors in brackets, natural logarithms) of G. swynnertoni, G. m. centralis and G. pallidipes, respectively, on different visual targets and in pyramidal traps. (DOCX) [file pntd.0006831.s002.docx]

**S1 Table.** Detransformed mean daily landing rates and catches (with transformed means ± standard errors in brackets, natural logarithms) of *G. swynnertoni*, *G. m. centralis* and *G. pallidipes*, respectively, on different visual targets and in pyramidal traps.

| **Device** | **Colour** | ***G. swynnertoni*** | ***G. pallidipes*** | | ***G. m. centralis*** | |
| --- | --- | --- | --- | --- | --- | --- |
|  |  | Tanzania | | | DR Congo | Angola |
| Pyramidal trap | blue/black |  | |  | 8.9 (2.18 ± 0.208) | 1.8 (0.59 ± 0.125) |
| Pyramidal trap with adhesive film | blue/black |  | |  | 13.1 (2.57 ± 0.140) | 4.6 (1.53 ± 0.124) |
| 1 m² square target | blue/black | 314.0 (5.75 ± 0.074) | | 104.5 (4.65 ± 0.159) |  |  |
| 1 m² horizontal oblong target | blue/black |  | |  | 17.9 (2.88 ± 0.094) | 4.8 (1.57 ± 0.202) |
| 0.5 m² horizontal oblong target | blue/black | 280.7 (5.64 ± 0.160) | | 57.9 (4.06 ± 0.267) | 19.1 (2.95 ± 0.122) | 4.4 (1.47 ± 0.162) |
|  | all-blue | 172.2 (5.15 ± 0.127) | | 60.7 (4.11 ± 0.242) | 11.0 (2.40 ± 0.140) | 4.0 (1.38 ± 0.113) |
| 0.5 m² square target | blue/black | 173.2 (5.15 ± 0.121) | | 45.2 (3.81 ± 0.197) | 15.3 (2.73 ± 0.112) | 3.9 (1.35 ± 0.142) |
|  | all-blue | 100.4 (4.61 ± 0.122) | | 34.0 (3.53 ± 0.262) |  |  |
