# Supplementary material for: Standardising visual control devices for Tsetse: East and Central African Savannah species Glossina swynnertoni, Glossina morsitans centralis and Glossina pallidipes
Source: PLoS Negl Trop Dis. 2018 Sep 25;12(9):e0006831. doi: 10.1371/journal.pntd.0006831 (PMC6173441; doi:10.1371/journal.pntd.0006831)
Supplement: S2 Table — Landing rate distributions of G. swynnertoni, G. m. centralis and G. pallidipes according to sex and substrate colour on visual targets and the pyramidal trap. (DOCX) [file pntd.0006831.s003.docx]

**S2 Table.** Landing rate distribution of three tsetse species according to sex and substrate colour on visual targets and the pyramidal trap.

| **Devices (and species)** |  |  |  |  |  |  |  |  |  |  |
| --- | --- | --- | --- | --- | --- | --- | --- | --- | --- | --- |
| ***G. swynnertoni*** |  | **daily catch** | **%male** | **%female** | **% on blue** | **% on black** | **males on blue** | **females on blue** | **males on black** | **females on black** |
| 1 m² square target | blue/black | 314.0 | 37% | 63% | 78% | 22% | 75% | 80% | 25% | 20% |
| 0.5 m² horizontal oblong | blue/black | 280.7 | 40% | 60% | 75% | 25% | 75% | 75% | 25% | 25% |
|  | all-blue | 172.2 | 38% | 62% | N/A | N/A | N/A | N/A | N/A | N/A |
| 0.5 m² square target | blue/black | 173.2 | 35% | 65% | 78% | 22% | 80% | 78% | 20% | 22% |
|  | all-blue | 100.4 | 33% | 67% | N/A | N/A | N/A | N/A | N/A | N/A |
| ***G. pallidipes*** |  |  |  |  |  |  |  |  |  |  |
| 1 m² square target | blue/black | 104.5 | 27% | 73% | 77% | 23% | 75% | 78% | 25% | 22% |
| 0.5 m² horizontal oblong | blue/black | 57.9 | 34% | 66% | 79% | 21% | 81% | 77% | 19% | 23% |
|  | all-blue | 60.7 | 32% | 68% | N/A | N/A | N/A | N/A | N/A | N/A |
| 0.5 m² square target | blue/black | 45.2 | 33% | 67% | 72% | 28% | 67% | 74% | 33% | 26% |
|  | all-blue | 34.0 | 32% | 68% | N/A | N/A | N/A | N/A | N/A | N/A |
| ***G. m. centralis* DRC** |  |  |  |  |  |  |  |  |  |  |
| Pyramidal trap | blue/black | 8.9 | 26% | 74% | N/A | N/A | N/A | N/A | N/A | N/A |
| Pyramidal trap with adhesive film | blue/black | 13.1 | 22% | 78% | 68% | 32% | 62% | 70% | 38% | 30% |
| 1 m² horizontal oblong | blue/black | 17.9 | 21% | 79% | 67% | 33% | 69% | 67% | 31% | 33% |
| 0.5 m² horizontal oblong | blue/black | 19.1 | 23% | 77% | 65% | 35% | 61% | 66% | 39% | 34% |
|  | all-blue | 11.0 | 18% | 82% | N/A | N/A | N/A | N/A | N/A | N/A |
| 0.5 m² square target | blue/black | 15.3 | 19% | 81% | 62% | 38% | 55% | 64% | 45% | 36% |
| ***G. m. centralis* Angola 2014** |  |  |  |  |  |  |  |  |  |  |
| Pyramidal trap | blue/black | 1.8 | 26% | 74% | N/A | N/A | N/A | N/A | N/A | N/A |
| Pyramidal trap with adhesive film | blue/black | 4.6 | 29% | 71% | 47% | 53% | 50% | 45% | 50% | 55% |
| 1 m² horizontal oblong | blue/black | 4.8 | 32% | 68% | 49% | 51% | 57% | 46% | 43% | 54% |
| 0.5 m² horizontal oblong | blue/black | 4.4 | 43% | 53% | 64% | 36% | 61% | 66% | 39% | 34% |
|  | all-blue | 4.0 | 49% | 51% | N/A | N/A | N/A | N/A | N/A | N/A |
| 0.5 m² square target | blue/black | 3.9 | 34% | 66% | 58% | 42% | 64% | 55% | 36% | 45% |
| ***G. m. centralis* Angola 2010** |  |  |  |  |  |  |  |  |  |  |
| 1 m² square target unabaited | blue/black | 52.4 | 29% | 71% | 71% | 29% | 71% | 72% | 29% | 28% |
| 1 m² square target with POCA | blue/black | 78.8 | 30% | 70% | 65% | 35% | 69% | 64% | 31% | 36% |
