# Supplementary material for: Standardising visual control devices for Tsetse: East and Central African Savannah species Glossina swynnertoni, Glossina morsitans centralis and Glossina pallidipes
Source: PLoS Negl Trop Dis. 2018 Sep 25;12(9):e0006831. doi: 10.1371/journal.pntd.0006831 (PMC6173441; doi:10.1371/journal.pntd.0006831)
Supplement: S3 Table — Detransformed mean daily landing rates and catches (with transformed means ± standard errors in brackets, natural logarithms) of G. m. centralis on a blue-black visual target, a clear target made of adhesive film, and in different traps, with and without the POCA bait. (DOCX) [file pntd.0006831.s004.docx]

**S3 Table.** Detransformed mean daily landing rates and catches (with transformed means ± standard errors in brackets, natural logarithms) of *G. m. centralis* on a blue-black visual target, a clear target made of adhesive film, and in different traps, with and without the POCA bait.

| **Device** | **Colour** | ***G. m. centralis* counts** | |
| --- | --- | --- | --- |
|  |  | Unbaited | POCA-baited |
| Pyramidal trap | blue/black | 11.1 (2.40 ± 0.276) | 27.1 (3.30 ± 0.222) |
| Epsilon trap | all-blue | 8.8 (2.17 ± 0.231) | 10.8 (2.37 ± 0.267) |
| 1 m² square target | blue/black | 52.4 (3.96 ± 0.129) | 78.8 (4.37 ±0.151) |
| 1 m² square adhesive film | clear | 6.1 (1.80 ± 0.184) | 8.8 (2.17 ± 0.206) |
